# Upregulation of LINC01503 promotes cervical cancer progression by targeting the miR-615-3p/CCND1 axis

**DOI:** 10.7150/jca.54148

**Published:** 2021-06-01

**Authors:** Jing Feng, Fang-yuan Gao, Yan-ying Li, Xi-yun Xiao, Jie Xi

**Affiliations:** Department of Gynecology, Cangzhou Central Hospital, Hebei, 061001, China.

**Keywords:** cervical cancer, LINC01503, miR-615-3p, CCND1

## Abstract

Mounting evidence indicates that long non-coding RNAs influence the progression of cervical cancer, but the precise function of LINC01503 in the pathogenesis of the disease remains unknown. Here, we found higher levels of LINC01503 in cervical cancer tissues. High LINC01503 expression was associated with enhanced progression of cervical cancer as indicated by advanced FIGO stage, increased metastasis of tumor cells to lymph nodes, and invasion into deeper cervical tissues. LINC01503 inhibition markedly suppressed the invasion and proliferative ability of tumor cells. Mechanistically, LINC01503 was demonstrated to negatively modulate the expression of miR-615-3p in cervical cancer. *CCND1* was found to be a target of miR-615-3p. Rescue experiments indicated that LINC01503 inhibition suppressed the invasion and proliferative ability of the tumor cells, a phenomenon that was reversed following miR-615-3p inhibition or *CCND1* overexpression. Collectively, these data indicate that LINC01503 enhances the progression of cervical cancer cells via interaction with miR-615-3p/*CCND1* axis.

## 1. Introduction

Cervical cancer (CC) is the second most prevalent malignancy affecting women globally 1, 2. Technological advancements have greatly improved diagnosis and treatment of CC, but the clinical outcomes remain poor as a result of metastasis/recurrence of the disease 3, 4. Therefore, it necessary to elucidate the pathogenesis and molecular mechanisms of CC and develop strategies to improve treatment outcomes.

Long non-coding RNAs (lncRNAs) belongs to a class of non-coding RNAs consisting of >200 nucleotides 5, 6. Multiple reports show that lncRNAs regulates various cellular processes consisting of proliferation, apoptosis, cell cycle progression, as well as invasion. LncRNAs are thought to exert their influence via chromatin remodeling, transcriptional control, and post-transcriptional regulation 7-9. Huang et al. reported that lncRNA MALAT1 can bind to the chromatin remodeling subunit *BRG1* to epigenetically enhance the progression of inflammation-induced liver cancer 10. Xiu et al. found that lncRNA TUG1 inhibition enhances radio-sensitivity of prostate cancer cells 11. A study by Hu et al., showed that lncRNA ROR sponged miR-138 and regulated *Mst1,* thereby exacerbating apoptosis of cardiomyocytes triggered by hypoxia or reoxygenation 12. The precise function of LINC01503 in the pathogenesis of CC remains unknown.

Here, we examined the role of LINC01503 in CC. Our findings confirm that this lncRNA facilitates the invasion as well as the proliferation of CC cells by interacting with the miR-615-3p/*CCND1* axis.

## 2. Materials and methods

### 2.1. Patients and specimens

Human CC tissues and matched normal tissues were collected from 43 patients treated at Cangzhou Central Hospital and were confirmed by 2 pathologists according to the guidelines of FIGO (International Federation of Gynecology and Obstetrics). None of the study subjects received any treatment (chemotherapy or radiotherapy) prior to surgery. All participants provided written informed consent. After collection, the specimens were briefly frozen in liquid nitrogen, and then preserved at -80°C. The ethics committee of Cangzhou Central Hospital approved the study. The clinical features of all the subjects are indicated in Table 1.

### 2.2. Cell culture and transfection

The CC cell lines consisting of SiHa, HeLa, C33A, and CaSki, as well as the Ect1/E6E7 normal human cervical cell line were procured from ATCC. Subsequently, the cells were grown in RPMI 1640 medium (Gibco) enriched with 10% fetal bovine serum (FBS) under a humidified environment with 5% CO_2_ and 37°C.

siRNA against LINC01503 (si-LINC01503#1: 5′- UCGGAAUACCCACCUUUCUGGUAAU-3′; si-LINC01503#2: 5′- UGACAAGUGUGUACCUACGUGUCAG-3′; si-LINC01503#3: 5′- CAUGACCGUGUGGAGAAAGUUCUUU-3′) and a non-targeting siRNA (si-NC: 5′-AATTCTCCGAACGTGTCACGT-3′) were supplied by GenePharma (Shanghai, China). RiboBio (Guangzhou, China) supplied the miR-615-3p mimics/inhibitor and matched controls. *CCND1* expression plasmid was constructed in a pcDNA3.1 vector (Thermo Fisher Scientific, Waltham, MA, USA). Lipofectamine 2000 was employed for cell transfection as described by the manufacturer.

### 2.3. Cell proliferation assessment

The CCK-8 kit (Dojindo, Kumamoto, Japan) was employed to explore cell proliferation. In brief, transfected CC cells suspected in 500µL of media were cultured in 96-well plates for 24, 48, or 72 hours. 10μL of CCK8 solution was introduced into each well and cultured for further 2 hours. Subsequently, a microplate reader was employed to determine the absorbance at 450nm.

Colony formation assays were done by seeding 5000 transfected CC cells into 6-well plates. Subsequently, the cells were cultured for 14 days, followed by fixation with methanol for 30 minutes. Afterwards, a staining step was conducted using by 0.1% crystal violet before counting colony numbers.

### 2.4. Transwell invasion assay

Inoculation of the transfected cells suspended in serum-free medium into the upper chamber of the transwell membrane (BD Biosciences) was performed. The medium containing 10% FBS was put in the bottom chamber. 24 hours later, the cells were stained in the dark with crystal violet stain (Sigma, MO, USA), after which the cells were counted.

### 2.5. Gene expression studies

TRIzol reagent (Thermo Fisher Scientific) was employed to isolate RNA from the cells as per the protocols described by the manufacturer. The RNA was converted into cDNA with a cDNA Synthesis kit (Revert Aid First Strand, Fermentas, Canada). PCR was done using SYBR Premix Ex Taq II kit (Takara, Tokyo, Japan) on a CFX96 Real-Time PCR machine (Biorad) using the following program: 95°C for 6 minutes (denaturation); and 40 cycles of 94°C for 30 seconds (initiation), 60°C for 30 seconds (annealing), and 73°C for 1.5 minutes (elongation). Relative gene expression was determined via the 2^-ΔΔCt^ approach. U6 along with GAPDH were served as the internal control.

### 2.6. Western blot assay

RIPA buffer (Sigma) enriched with 1mM PMSF was used to lyse the cells. A BCA assay kit (Thermo Fisher Scientific) was utilized to measure the protein concentration. Next, the proteins were resolved with SDS-PAGE, and then blotted onto PVDF membranes. After that, 5% skimmed milk was utilized to prevent non-specific binding on the membranes for 1 hour, and overnight incubation was conducted at 4°C with anti-CCND1 primary antibody. The membranes were rinsed and incubated with HRP-labelled goat anti-rabbit secondary antibody (Abcam) and incubated at room temperature. The signal was then developed using enhanced chemiluminescence reagent (Advansta, MO, USA). Finally, the ImageJ was employed to quantify the proteins and the GAPDH gene utilized as the normalization control.

### 2.7. Dual-luciferase reporter analysis

Wildtype (WT) and mutant (MUT) LINC01503 or *CCND1* 3'UTR were amplified, then cloned into the pmirGlO luciferase reporter vector (Promega, Madison, WI, USA) to create WT-LINC01503, WT-*CCND1*, MUT-LINC01503, and MUT-*CCND1* vectors. Cells were co-introduced into these reporters and miR-615-3p (or miR-NC) via co-transfection and cultured for 48 hours. Dual-luciferase assays (Promega) were then carried out to determine the luciferase activity and normalized to Renilla luciferase activity.

### 2.8. RIP assay

The Magna RNA binding protein immunoprecipitation kit (Millipore, Billerica, MA, USA) was utilized for the RIP assay. In summary, whole-cell lysates were prepared using RIP buffer, and then incubation with magnetic beads hybridized with human anti-Ago2 antibody or normal mouse IgG as a negative control performed. The sample was then incubated with proteinase K, followed by RNA immunoprecipitation. The immunoprecipitated RNA was purified and analyzed by RT-qPCR to quantify LINC01503 and miR-615-3p expression.

### 2.9. Nude mouse model *in vivo*

The Animal Care and Use Committee of Cangzhou Central Hospital approved all the mice experiments. We subcutaneously administered 2×10^6^ stably transfected cells into BALB/C 4-week-old female nude mice weighing 18-25g (n=4 for each group). Tumor volume was monitored using a caliper weekly from the 7^th^ day after injection. Tumor volume was computed via the formula: length × width^2^ × 0.5. The animals were euthanized six weeks after xenografting using cervical dislocation, the tumors weights were measured for downstream analyses.

### 2.10. Bioinformatics analysis

The present study determined LINC01503 expression into the GEPIA dataset to explore its differential expression and influence on the prognosis of individuals with CESE, OV, UCEC, and UCS. TargetScan, miRcode, LncBase v.2 and Starbase V2.0 were used to investigate the potential target of LINC01503. TargetScan, miRWalk as well as miRTarBase were employed in predicting the prospective target gene of miR-615-3p.

### 2.11. Data analysis

Data analysis was conducted with the SPSS version 20.0. All data are indicated as mean ± standard deviation (SD). Student's t-test was employed to determine differences between 2 groups. One-way ANOVA followed by Tukey test (post-hoc) was utilized to analyze differences across multiple groups. P<0.05 represented statistical significance.

## 3. Results

### 3.1. LINC01503 expression is significantly upregulated in CC cells and tissues

**Figure 1:**
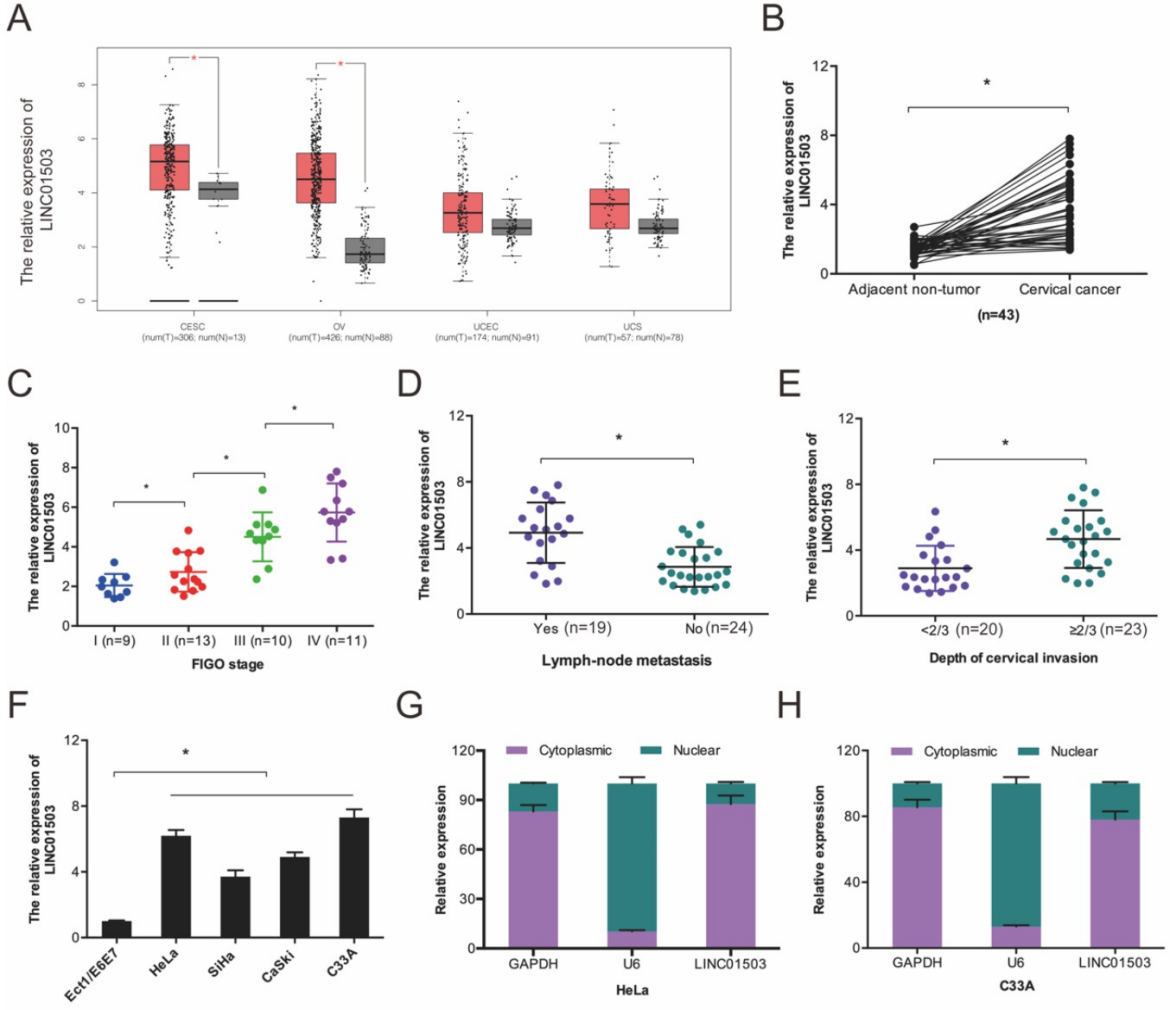
LINC01503 expression is enhanced in CC. (A) LINC01503 expression LINC01503, as determined on the GEPIA database. (B) RT-qPCR analysis of LINC01503 expression in 43 paired CC tissues. (C-E) High levels of LINC01503 positively linked to advanced FIGO stage, lymph node metastasis, as well as depth of cervical invasion in patients. (F) RT-qPCR analysis of LINC01503 expression in CC cells. (G, H) The subcellular localization of LINC01503 as analyzed by subcellular fractionation assay. OV: Ovarian serous cystadenocarcinoma; CESE: Cervical squamous cell carcinoma and endocervical adenocarcinoma; UCEC: Uterine Corpus Endometrial Carcinoma; UCS: Uterine Carcinosarcoma. *P ≤0.05.

We first evaluated LINC01503 expression in the GEPIA database and found that LINC01503 was higher in CC tissues relative to the normal tissues (Figure 1A). Next, we compared LINC01503 expression between 43 CC tissues and paired healthy cervical tissues. According to the RT-qPCR assay, LINC01503 expression was higher in CC tissues in contrast with the controls (Figure 1B), and its high expression was associated with enhanced progression of CC as indicated by advanced FIGO stage, lymph-node metastasis, as well as depth of cervical invasion (Figure 1C-E). Further analysis demonstrated that LINC01503 expression in CC cell lines (SiHa, C33A, HeLa, as well as CaSki) were remarkably higher in compared to normal cervical cell line Ect1/E6E7 (Figure 1F). Intracellular localization revealed that LINC01503 was mainly expressed in the cytoplasm (Figure 1G-H). Collectively, these observations suggested that LINC01503 has a critical role in CC progression.

### 3.2. LINC01503 promotes CC growth and metastasis

**Figure 2:**
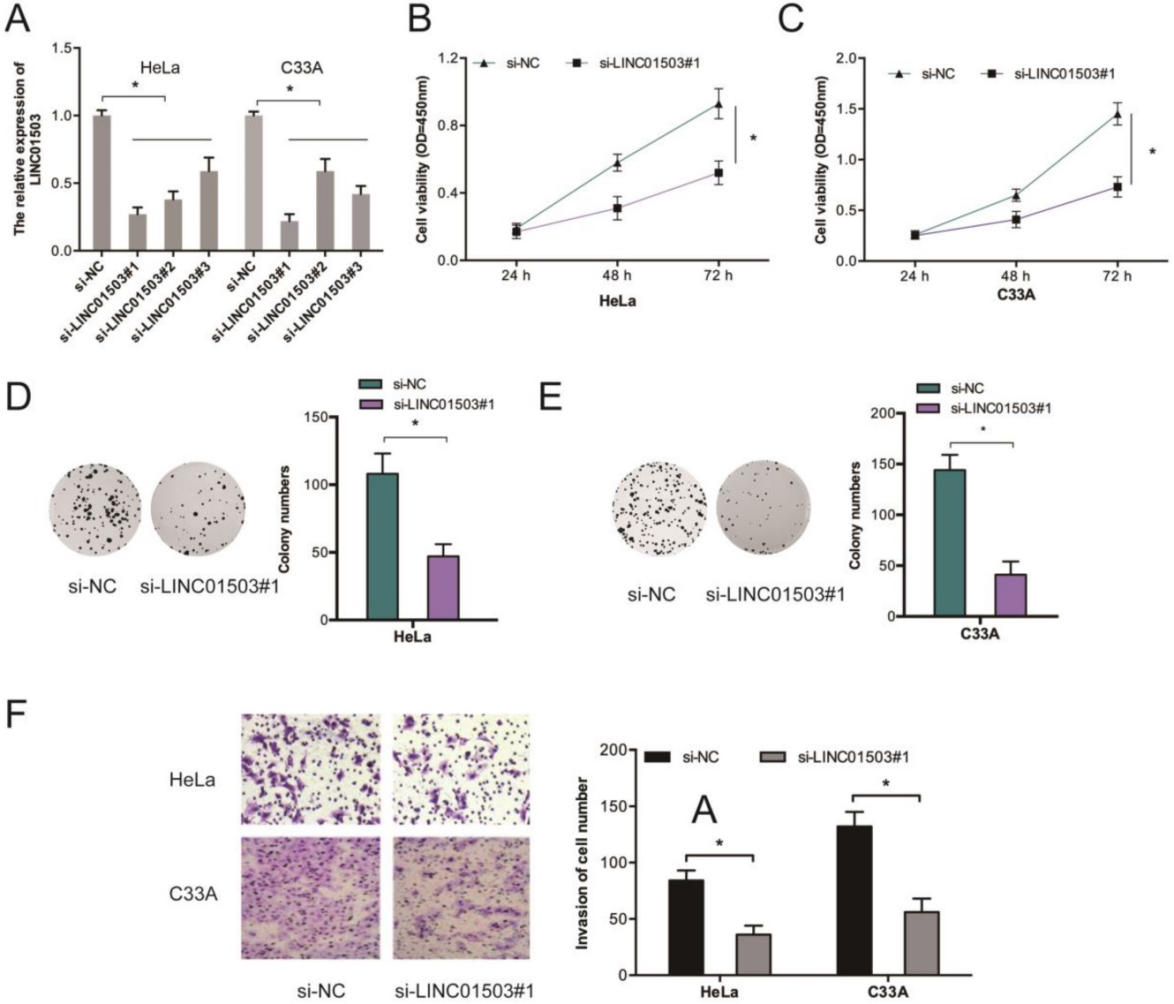
LINC01503 enhances the invasion and proliferative ability of CC cells. (A) HeLa, C33A cells were infected with si-NC or si-LINC01503 vectors. (B-E) LINC01503 inhibition suppresses the viability of CC cells as demonstrated by CCK-8 as well as colony formation assays. (F) LINC01503 KD decreases CC cell invasion. *P ≤0.05.

To further assess the influence of LINC01503 on CC growth and metastasis, we silenced LINC01503 expressions in HeLa as well as C33A cells (Figure 2A). Results showed that LINC01503 knockdown (KD) suppressed the proliferative ability of HeLa and C33A cells relative to si-NC KD cells (Figure 2B-E). Transwell assay showed that LINC01503 inhibition suppressed cell invasion (Figure 2F).

**Figure 3:**
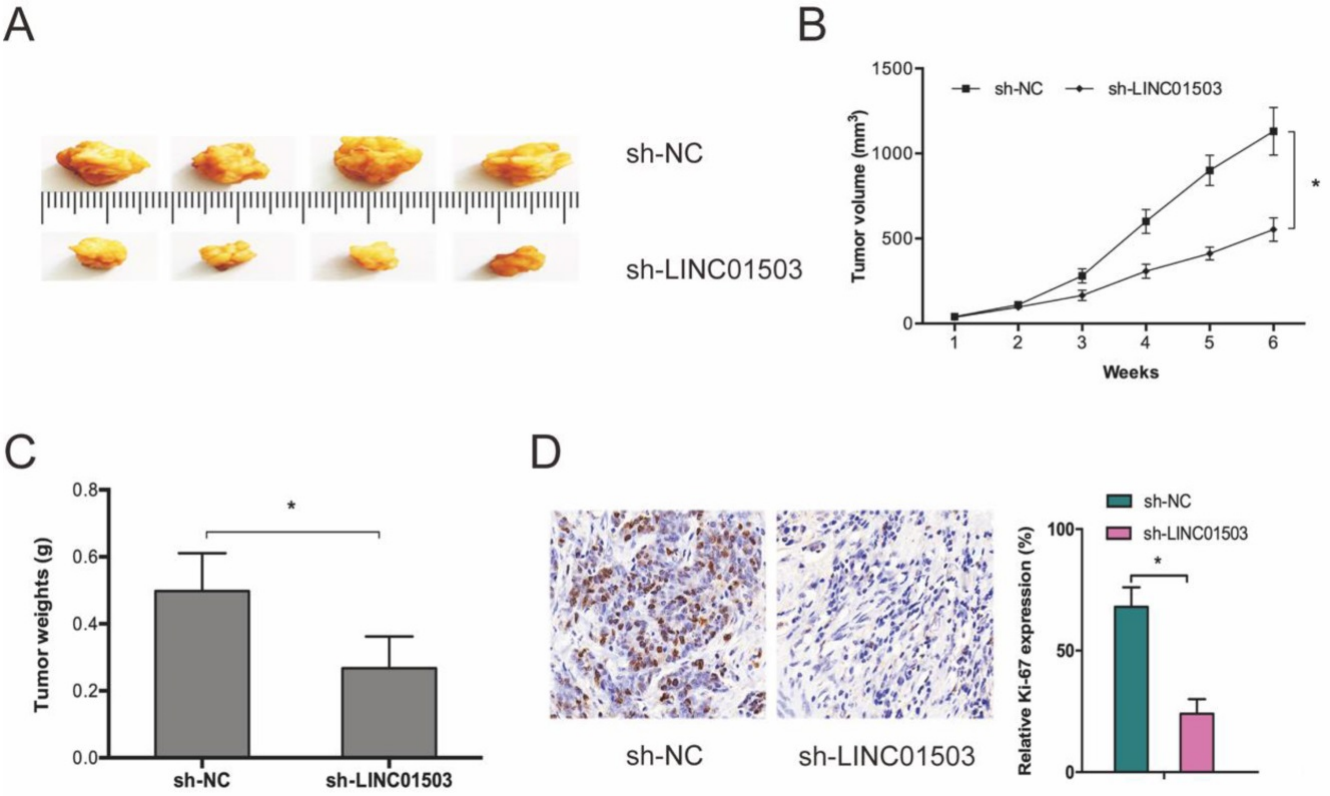
LINC01503 promotes CC growth *in vivo*. (A-C) LINC01503 suppression reduces tumor volume and weight. (D) Assessment of Ki67 expression in tumor tissue by IHC assay. *P ≤0.05.

Next, we subcutaneously xenografted stable sh-LINC01503 into nude mice and monitored tumor growth weekly. It was found that LINC01503 inhibition significantly suppressed tumor growth* in vivo* (Figure 3A-C). As expected, IHC analysis showed that sh-LINC01503 decreased Ki67 expressions in tumor tissues of mice (Figure 3D). Together, these data show that LINC01503 might have an oncogenic role in CC.

### 3.3. LINC01503 acts as a ceRNA by binding to miR-615-3p

**Figure 4:**
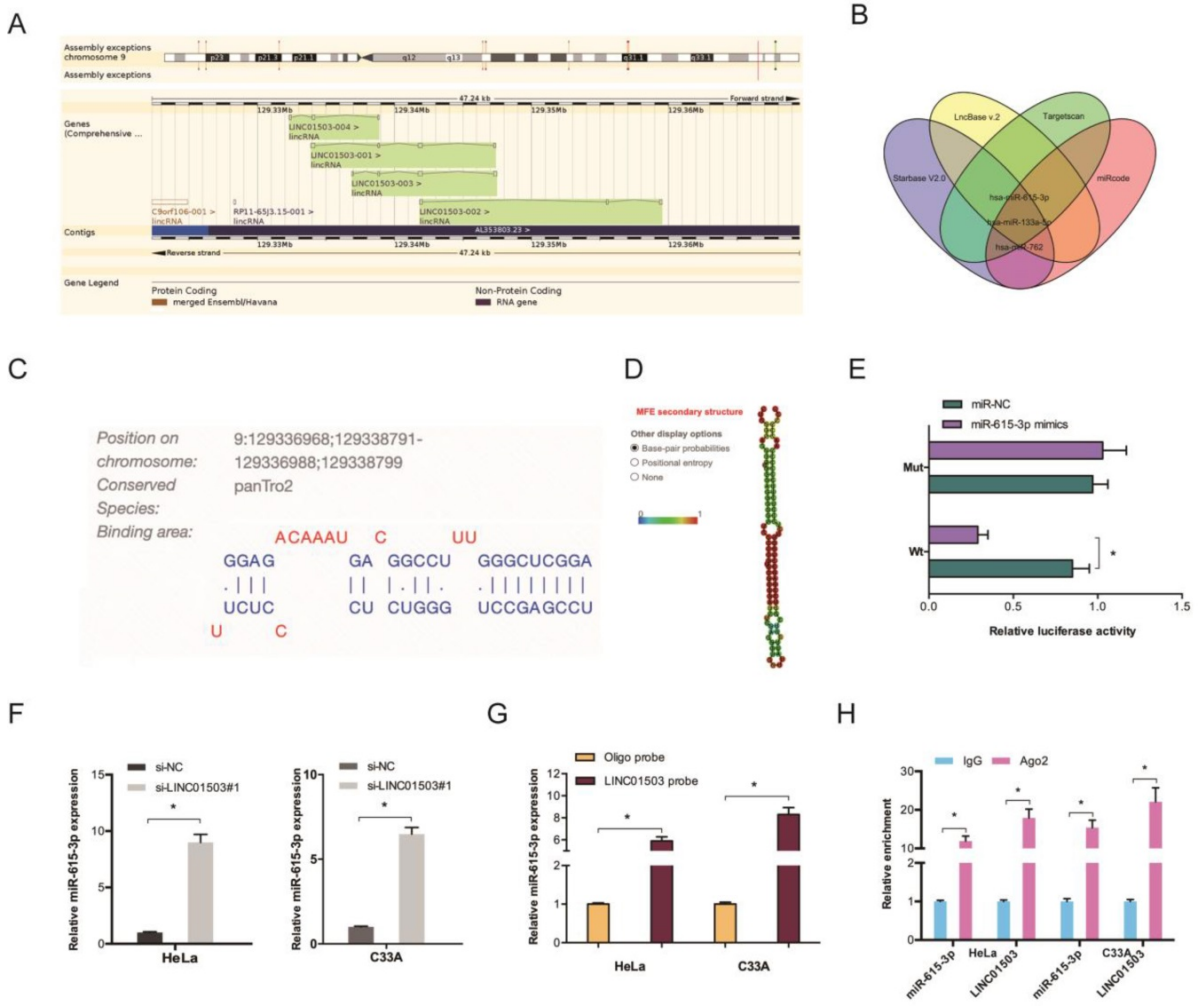
LINC01503 acts as ceRNA by binding to miR-615-3p. (A) Information of LINC01503. (B) Venn diagram showing the potential targets of LINC01503. (C) The biding site of LINC01503 on miR-615-3p. (D) The secondary structure of miR-615-3p. (E) miR-615-3p mimic reduces the fluorescence activity of LINC01503-WT group. (F) Silencing of LINC01503 significantly increased miR-615-3p expression (G, H) RNA pull-down and RIP analysis validated the association of LINC01503 with miR-615-3p in CC. *P ≤0.05.

LncRNAs regulate cellular processes by functioning as ceRNA and sponging miRNAs. To explore the role of LINC01503, Starbase V2.0, LncBase v.2, Targetscan, and miRcode were used to identify the targets of LINC01503 (Figure 4A-B). The binding site for LINC01503 on miR-615-3p 3'UTR is shown in Figure 4C-D. Luciferase assay indicated that miR-615-3p mimics suppressed the fluorescence activity of LINC01503-WT reporter (Figure 4E). The RT-qPCR analysis showed that LINC01503 knockdown markedly enhanced miR-615-3p expression in CC cell lines (Figure 4F). RNA pull-down and RIP analysis further confirmed the relationship of LINC01503 with miR-615-3p in CC (Figure 4G-H).

**Figure 5:**
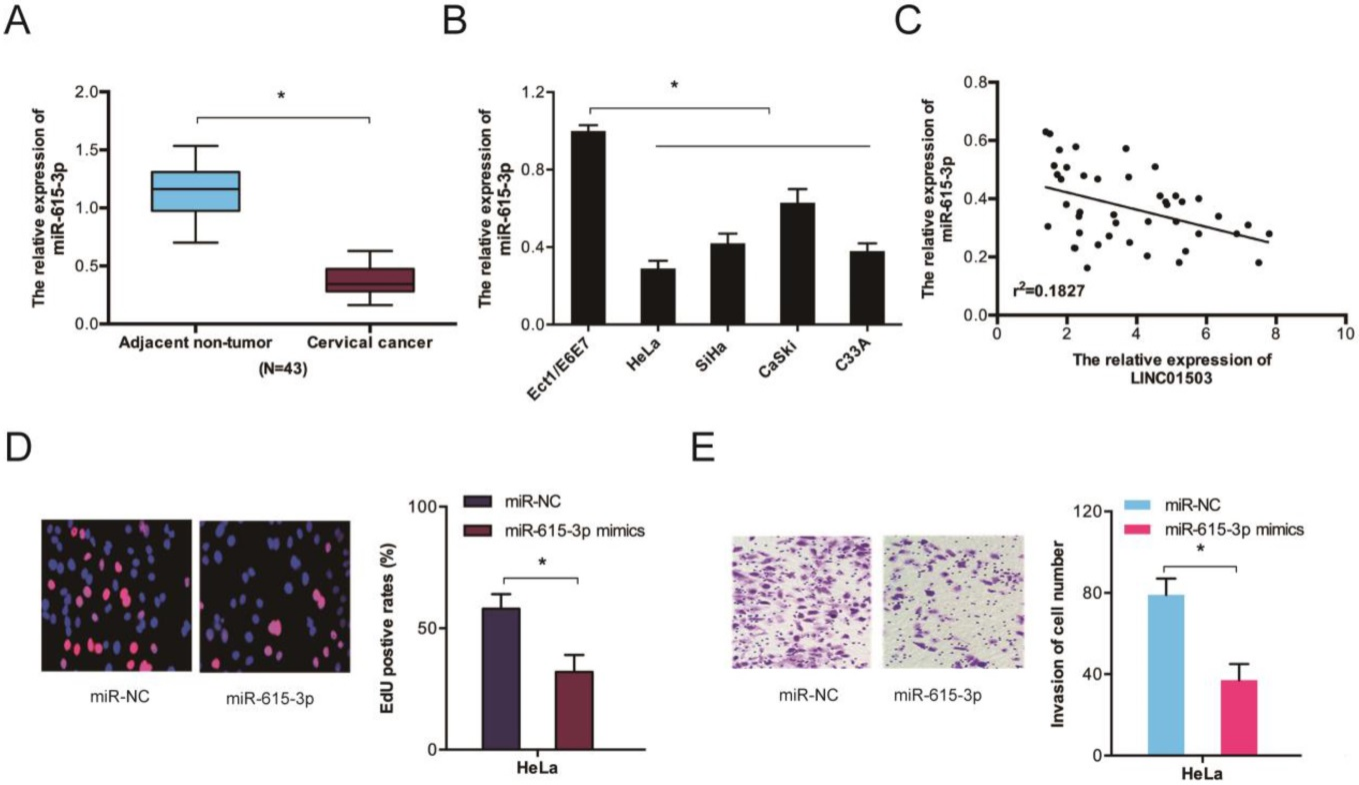
The function of miR-615-3p in CC. (A, B) RT-qPCR determination of miR-615-3p expression in CC tissues as well as cells. (C) Crosstalk between LINC01503 expression and miR-615-3p in CC tissues. (D, E) Edu and transwell invasion assays verified that miR-615-3p overexpression reduces CC cell viability and invasion. *P ≤0.05.

We further assessed miR-615-3p expression in CC by RT-qPCR and observed that the levels of miR-615-3p were remarkably lower in CC tissues and cell lines (Figure 5A-B). Correlation analysis indicated a crosstalk between LINC01503 and miR-615-3p in CC tissues (Figure 5C). Evaluation of the cellular functions of miR-615-3p using Edu and transwell invasion assays showed that the proliferative as well as the invasion ability of HeLa cells were inhibited when miR-615-3p was upregulated (Figure 5D-E). Together, these data indicate that LINC01503 might regulate the progression of CC by interacting with miR-615-3p.

### 3.4. LINC01503 positively modulates CCND1 by sponging miR-615-3p

**Figure 6:**
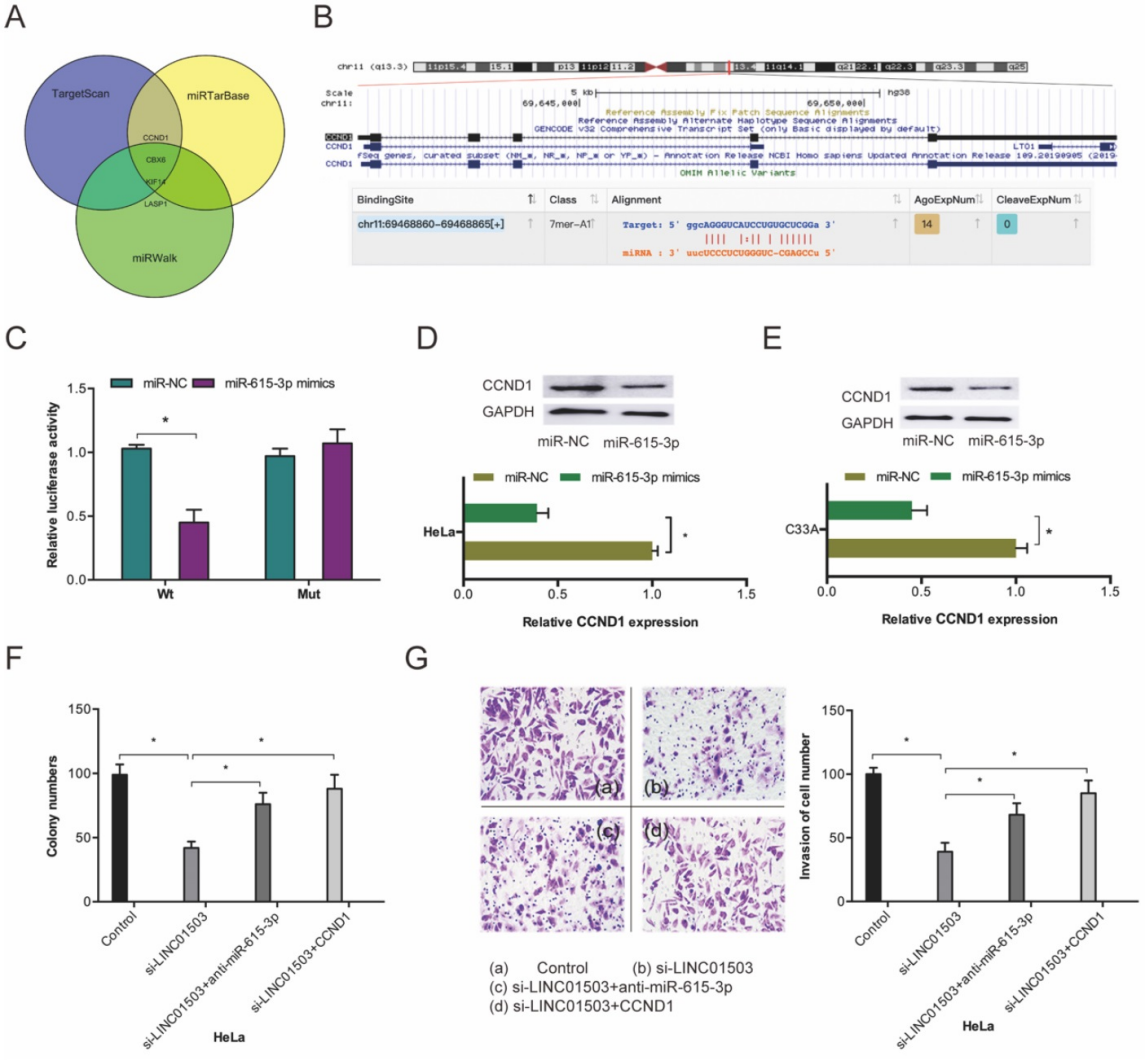
miR-615-3p targets *CCND1* in CC. (A) Venn diagrams showing the potential miR-615-3p targets. (B) The biding site of *CCND1* on miR-615-3p. (C) Relative luciferase enzyme activity was assessed in CC cells co-transfected with *CCND1*-WT, *CCND1*-MUT, miR-NC, or miR-615-3p mimic. (D, E) miR-615-3p overexpression reduces *CCND1* protein levels in CC cells. (F, G) miR-615-3p inhibitors or *CCND1* overexpression abolished the regulation of si-LINC01503 on CC cell proliferation along with invasion. *P ≤0.05.

To investigate the mechanism of miR-615-3p in CC cells, we conducted target prediction analysis on TargetScan, miRTarBase, and miRWalk databases and identified *CCND1* as a promising miR-615-3p target (Fig. 6A-B). Dual-luciferase reporter assessment found that miR-615-3p mimics remarkably repressed luciferase enzyme activity in *CCND1*-WT (Figure 6C). Next, we assessed the influence of miR-615-3p on *CCDN1* expression. Western blot assay demonstrated that miR-615-3p overexpression reduced the expression levels of *CCND1* in HeLa and C33A cells (Figure 6D-E).

Furthermore, we performed rescue assays to verify the involvement of LINC01503/miR-615-3p/*CCND1* axis in CC progression. Colony formation and transwell invasion assays revealed that miR-615-3p inhibitors or *CCND1* upregulation significantly obliterated the suppression of Hela cell proliferation as well as invasion by si-LINC01503 (Figure 6F-G). This indicating that LINC01503 promotes the progression of CC cells by interacting with the miR-615-3p/*CCND1* axis.

## 4. Discussion

Accumulating evidence indicates that lncRNAs influences the progression of diseases 13, 14. The recently discovered lncRNA, LINC01503 is located on chromosome 9:129,336,879-129,347,464 in humans. It has been reported that LINC01503 influences tumorigenesis. For instance, Xie et al. reported that LINC01503 is highly expressed in squamous cell carcinoma and promotes proliferation, migration, invasion, as well as the growth of tumor xenografts 15. Ding et al. showed that LINC01503 enhances the invasion and proliferation of tumor cells by regulating Wnt signaling in gastric cancer 16. Lu et al. found that LINC01503 facilitates progression of colorectal cancer via the miR-4492/*FOXK1* axis 17. Moreover, Peng et al. found that LINC01503 enhances the progression of tumor cells by modulating miR-342-3p /FXYD3 axis in CC 18. However, the roles of LINC01503 in CC development and progression remains unclear.

Here, we established that LINC01503 was upregulated in CC cells as well as tissues and that this associated with advanced clinical characteristics. Additionally, we observed that LINC01503 knockdown inhibited CC progression by suppressing the metastatic and proliferative abilities of tumor cells, but the precise mechanism is yet to be revealed.

LncRNAs have been found to influence tumorigenesis by sponging miRNAs 19. Several studies have found that lncRNA functions as ceRNAs to sponge miRNAs in CC. Zhu et al. revealed that *TUG1* sponges miR-138-5p to promote CC progression 20. Yang et al. indicated that lncRNA OIP5-AS1 enhances the invasion and proliferative abilities of CC cells by upregulating *ITGA6* and sponging miR-143-3p 21. Here, we report that LINC01503 might function as a ceRNA for miR-615-3p in CC. LINC01503 suppression upregulated miR-615-3p in CC cells. Furthermore, using luciferase reporter, RNA pull-down, as well as RIP assays, we verified the cross talk between LINC01503 and miR-615-3p. Moreover, miR-615-3p was demonstrated to be poorly expressed in CC tissues and it suppressed tumor growth along with invasion *in vitro*. Collectively, our data demonstrates that miR-615-3p mediates the oncogenic function of LINC01503 in CC.

*CCND1*, along with cyclin-dependent kinases, modulates G1/S transition through Rb phosphorylation 22, 23. Accumulating evidence shows that *CCND1* influences a plethora of biological processes associated with the pathogenesis, as well as drug resistance in human cancers 24, 25. *CCND1* is therefore recognized as an oncogene in multiple cancer types consisting of lung cancer, osteosarcoma, and nasopharyngeal carcinoma et al 24-26. Nevertheless, the function of *CCND1* in CC is unknown. Here, we found that *CCND1* is a target of miR-615-3p in CC and is negatively modulated by miR-615-3p. We showed that higher levels of *CCND1* abolish the suppressive effects of LINC01503 KD on CC cell proliferation and invasion. Collectively, the results indicate that LINC01503 knockdown suppresses CC progression by interacting with the miR-615-3p/*CCND1* axis.

In conclusion, our data show that LINC01503 is upregulated in CC and its knockdown suppresses the invasion and proliferative ability of CC cells. Therefore, it modulates miR-615-3p expression, thereby suppresses *CCND1* expression. Our study highlights the therapeutic potential of targeting the LINC01503/miR-615-3p/*CCND1* axis in the treatment of CC.

## Figures and Tables

**Table 1:** The clinicopathological characteristics of cervical cancer patients.

Characteristics		Number
Age	<50	28
	≥50	15
Tumor size (cm)	<4	26
	≥4	17
Depth of cervical invasion	<2/3	20
	≥2/3	23
Histological type	Adenocarcinoma	13
	Squamous cell carcinoma	30
Lymph node metastasis	No	24
	Yes	19
Figo stage	I	9
	II	13
	III	10
	IV	11

## Abbreviations

CC: Cervical cancer
CESE: Cervical squamous cell carcinoma and endocervical adenocarcinoma
OV: Ovarian serous cystadenocarcinoma
UCEC: Uterine Corpus Endometrial Carcinoma
UCS: Uterine Carcinosarcoma
lncRNAs: Long non-coding RNAs
miRNAs: MicroRNAs
Mut: Mutant
WT: Wild type
UTR: Untranslated region
